# Nonsuicidal Self-Injury and Suicidal Beliefs in Adolescent Inpatient

**DOI:** 10.3390/medicina60050796

**Published:** 2024-05-11

**Authors:** Jelena Kostic, Olivera Žikić, Vladimir Djordjević, Aleksandra Ristić, Žilijeta Krivokapić

**Affiliations:** 1 Medical College, University of Niš, 18000 Niš, Serbia; 2 Mental Health Protection University Clinical Centre Niš, 18000 Niš, Serbia; 3 The College of Applied Health Sciences Ćuprija, 35230 Ćuprija, Serbia

**Keywords:** nonsuicidal self-injury, adolescent inpatient, suicidal beliefs

## Abstract

*Background and Objectives:* Although nonsuicidal self-injury (NSSI), by definition, excludes suicidal intent, numerous studies show associations between NSSI and suicidal phenomena in clinical and outpatient adolescent samples. Given the growing interest in the relationship between NSSI and suicidal phenomena, the present study aimed to investigate the relationship between NSSI and suicidal beliefs in adolescent psychiatric inpatients. *Materials and Methods:* The study sample included 50 adolescent inpatients at a specialized facility, with a mean age of 15.44 ± 1.39, who fulfilled DSM-5 criteria for NSSI. For study purposes, we use the Ottawa Self-Injury Inventory (OSI) and Brief Suicide Cognitions Scale (B-SCS). Statistical data processing was performed in the R software 4.3.0 (R Core Team, Vienna, Austria). *Results*: Of all NSSI functions, the Internal ER function score was the highest (18.72 ± 7.08), followed by External ER (8.10 ± 3.11), Social Influence (5.88 ± 5.37), and Sensation Seeking (3.44 ± 2.98). The mean Craving (C) score was 14.06 ± 7.51. The mean value of the B-SCS score was 19.54 ± 5.24. It was found that the B-SCS score is significantly related to Internal ER (r = 0.441, *p* < 0.001) and Craving (r = 0.297, *p* = 0.036). The multivariable model shows that internal ER function and participants’ age are significantly related to the B-SCS score. *Conclusion*: Despite the limitations of the study, it is emphasized that cognitions occurring across the fluid suicidal belief system alone do not fully capture the complexity of suicide, but assessing the suicidal belief system in NSSI inpatient adolescents could nevertheless provide helpful information for identifying individuals who may have an elevated vulnerability to experiencing suicidal ideas and behaviors over time.

## 1. Introduction

Nonsuicidal self-injury (NSSI) is defined as repeated (at least five days in a year) socially unacceptable acts of deliberate self-harm to one’s body tissue without suicidal intent [1]. There are many types of NSSI behaviors, including cutting, biting, burning, hitting, scratching, etc. The onset of NSSI is noted in early adolescence, with rates increasing in adolescence and generally ceasing in young adulthood [2]. A relatively large number of adolescents in the general population experiment with NSSI; however, only 1.5% to 6.7% exhibit recurrent, intentional self-injury that meets DSM-5 criteria [3]. In clinical samples of adolescents, prevalence rates of NSSI are as high as 60% for single NSSI and approximately 50% for repeated NSSI [4].

While previously, the phenomenon of NSSI was attributed to borderline personality disorder (BPD), today it is known that it also occurs in the absence of borderline personality disorder, i.e., within the framework of other psychiatric disorders, which support conceptualizations of NSSI as transdiagnostic phenomena [5].Research on adolescents has demonstrated that NSSI is motivated by a variety of different functions that can be categorized as intrapersonal (based on emotion regulation) or interpersonal (based on social processes), which are not mutually exclusive [6]. Recent meta-analyses by Wolf et al. [7] show that emotional dysregulation is one of the most stable correlates of NSSI, regardless of age, gender, or type of sample (clinical or outpatient).

Although NSSI, by definition, excludes suicidal intent, studies show associations between NSSI and suicidal phenomena in clinical and outpatient samples, which significantly increases the risk of suicide [8,9,10,11]. Nock et al. [10] found that 70% of adolescents who had recently committed NSSI reported at least one suicide attempt, and 55% reported multiple attempts. Some authors [12] describe NSSI as a “gateway” to suicide. In a study by Glenn et al. [9] with a sample of 270 inpatient and outpatient adolescents with an average age of 15.54 years, it was found that the majority of adolescents reported both NSSI and suicidal ideation, and one-third reported both NSSI and suicide attempts in their lifetime. The research findings [9] suggest that the onset of NSSI thoughts and suicidal ideation may occur at the same time and before the first NSSI activity and that the pathways to NSSI and suicidal behavior may co-occur rather than occurring sequentially from nonsuicidal to suicidal self-harm.

Suicidal ideation, broadly defined as “thoughts of suicidal behavior” [13], refers to thinking or planning behavior with the explicit intent to die shortly, and suicidal ideation is thought to be a necessary precondition for suicidal behavior aimed at ending life [14]. Because considerable natural variability in suicidal ideation, negative affect, and individual history of self-harm has been shown, with hour-to-hour, day-to-day, and week-to- week variations that vary widely among the most vulnerable individuals [15], research attention has turned to theoretical models that assume that some individuals are more sensitized or “vulnerable” to suicidal behavior and that this vulnerability persists over time regardless of the individual’s specific psychiatric symptom profile. Fluid Vulnerability Theory (FVT) [16] uses the term acute suicidality episode, emphasizing that there is no continuous suicidal risk but that it should rather be seen as a recurrent, acute risk whose occurrence depends on the activation of the suicidal mode [17]. The activation of suicidal mode does not happen by chance. However, certain cognitive, emotional, physiological, and behavioral domains make some people more vulnerable, and cognitive sensitization can also be responsible for it. The cognitive domain includes variables such as problem-solving style, cognitive flexibility/rigidity, and suicide-related cognitive distortions (e.g., hopelessness, enmeshment, perceived distress). The broad spectrum of suicide-related cognitive distortions is referred to as the suicidal belief system [16], as it encompasses various combinations of perspectives and cognitive styles associated with the understanding of self, others, and the world. Suicidal belief systems are characterized by pervasive identity-based hopelessness and are captured by core beliefs about the self as unlovable, one’s emotional experiences as unbearable, and one’s life problems as unsolvable, leading to a persistent vulnerability to the occurrence of acute suicidal crises over time, secondary to internal and external triggers. The suicidal belief system has been found to significantly predict future suicidal behaviors, even when covarying out suicidal ideation [18].

Given the growing interest in the relationship between NSSI and suicidal phenomena, the present study aimed to investigate the relationship between NSSI and suicidal beliefs in adolescent psychiatric inpatients. The study aimed to collect information on the NSSI’s features and functions, assess the suicidal belief system, and examine the relationship between the NSSI’s features and functions and the suicidal belief system in the adolescent inpatient sample.

## 2. Subjects and Method

### 2.1. Participants and Procedure

The participants were recruited in the Child and Adolescent Psychiatry inpatient department at the Centre for Mental Health Protection University Clinical Centre Niš. The recruitment process lasted from September 2023 to January 2024. The study sample included fifty adolescents, forty-one females and nine males, aged 12 to 18 years. For all participants, there were data on non-suicidal self-injury (presence, type) in the last year and at any time during life (operational definitions according to DSM-5), and all participants fulfilled DSM-5 criteria for NSSI [1]. The assessment of NSSI was based on several sources of information (interview, medical history, heteroanamnesis, consultation reports, nursing documentation, and current examination status). The assessment of psychopathological symptoms was based on the profiles of internalizing (social withdrawal, anxiety, depression, emotional problems) and externalizing symptoms (aggression, impulsivity, rule violation, hyperactivity) by the supervising psychologist for each patient. Adolescents with a mental disability, a current or previous psychotic disorder, and acute substance abuse were excluded from this study.

### 2.2. Measures

The Ottawa Self-Injury Inventory [19] (OSI) is a self-report inventory that provides a comprehensive assessment of non-suicidal self-injury (NSSI) and includes both NSSI’s functions and addictive features as well as additional NSSI features. It contains quantitative (dichotomous, categorical, and continuous) and qualitative (open-ended) items. The OSI provides a four-factor structure of OSI functions that relate to four motivational categories underlying NSSI behaviors—Internal Emotion Regulation (Internal ER) with eight items (e.g., to relieve feelings of sadness or feeling “down”), Social Influence with nine items (e.g., to belong to a group), External Emotion Regulation (External ER) with three items (e.g., to relieve frustration), Sensation Seeking with four items (e.g., to experience a “high” like a drug high), and the single-factor structure of Addictive features (Craving C) with seven questions (e.g., the self-injurious behavior occurs more frequently than intended). The OSI is valid and reliable, with internal consistency values of 0.67 to 0.87 in a university sample of young adults, and is suitable for use with clinical samples of adolescents [20]. For research purposes, to determine the recent occurrence and frequency of NSSI, we used the OSI item “How often have you self-harmed in the last six months without the intention of killing yourself?” with a value range between 0 (not at all) and 4 (daily). For the OSI question “Have you thought about taking your own life (killing yourself) in the last year?”, a binary response was used as an option (presence or absence of suicidal thoughts in the last year). A binary response was also used for the item on lifetime suicide attempts “Have you ever tried to take your own life”. When interpreting the item “If you feel the urge to do something to yourself,” we considered the scores from 2 to 4. The first author contacted the instrument’s developer to inform her about the study’s use of it. Two authors translated the OSI questionnaire from English into Serbian, and the back-translation into English was formatted so that it was understandable for the study participants.

The Brief Suicide Cognitions Scale (B-SCS) [18] is a 6-item self-report scale that assesses the meaning of negative maladaptive perceptions of self and the world. The B-SCS assesses cognitions such as “unlovable”, “unbearable”, and “unsolvable”, which are influenced by a common underlying suicidal belief system and are captured by items in the scale (e.g., “I am completely unworthy of love”, “No one can help me solve my problems”). There are two items each for unlovability, intolerability, and unsolvability. Only one BSCS item mentions the word “suicide” (e.g., “Suicide is the only way to end this pain”). The items are rated on a 5-point Likert scale from 1 (strongly disagree) to 5 (strongly agree), indicating the extent to which the respondent agrees or disagrees with the statement. The point scale ranges from 6 to 30, with higher scores indicating a higher severity of suicidality. The B-SCS has shown acceptable predictive validity in the clinical setting, with internal consistency values of 0.84 to 0.90 [18]. The single total score of the B-SCS was used for the study. The author’s permission to use the scale in the study was obtained, and the scale was translated using the back-translation method and converted into a format conceptually equivalent to the original scale.

### 2.3. Data Analyses 

The data are presented in arithmetic mean, standard deviation, and absolute and relative numbers. The two sub-sample score values were compared using the *t*-test or Mann–Whitney test according to data distribution. Cronbach’s alpha was calculated to determine the reliability of the scales. For OSI, alpha = 0.849, B-SCS (0.853). Correlation analysis was used to assess the association of the B-SCS score with the investigated NSSI functions. The association of B-SCS score with demographic and other parameters was tested by multivariate regression analysis. The null hypothesis was tested with a significance threshold of *p* < 0.05. Statistical data processing was performed in the R software 4.3.0 (R Core Team, Vienna, Austria) [21].

### 2.4. Ethical Considerations

All participants and their parents/guardians were informed about the study’s purpose and gave their written consent to participate. Participation in the study was voluntary. The Ethics Committee of the University Hospital of Nis gave ethical approval for the study. 

## 3. Results

### 3.1. Sociodemographic Characteristics and Psychopathological Symptoms of the Sample

The sample comprised 50 adolescents, 82.0% (*n* = 41) females and 18.0% (*n* = 9) males. The mean age was 15.44 ± 1.39 years (min 12 years, max 18 years). Based on the psychopathological assessment, internalizing symptoms were found in 66.0% (*n* = 33) of the participants and externalizing symptoms in 34.0% (*n* = 17).

### 3.2. Descriptive Statistics for the Most Informative OSI Items Can Be Found in Table 1

In the study sample, 8.0% (*n* = 4) of participants had self-harmed between one and five times in the past six months, 28.0% (*n* = 14) reported monthly NSSI, 56.0% (*n* = 28) reported weekly NSSI, and 8.0% (*n* = 4) reported daily NSSI. Of all participants, 66% (*n* = 33) answered positively to the question, “Have you thought about taking your own life (killing youself?) in the past year?”, while 10.0% (*n* = 5) had a history of attempted suicide. The most commonly reported method of NSSI was cutting—48.0% (*n* = 24), scratching—24% (*n* = 12), piercing the skin with sharp objects—18% (*n* = 9), biting—14% (*n* = 7), and taking too much medication—4% (*n* = 2). The most commonly affected body parts were forearm/wrist—68% (*n* = 30), thigh/knee—52% (*n* = 26), hand/finger—28% (*n* = 14), abdomen—20 (*n* = 10), and face—18% (*n* = 9). The mean age at onset of NSSI was 12.96 ± 1.63. A total of 20% (*n* = 10) of adolescents had been treated by a doctor after self-injuring, and only 4% (*n* = 2) stayed in the emergency room overnight because of self-injury. Experiencing the urge to self-harm was rated most strongly as intrusive (2.16 ± 1.38), followed by distressing (2.14 ± 1.41) and comforting (1.64 ± 1.35). A total of 58.0% (*n* = 29) of participants stated that they “always” feel relief after self-harming, 32.0% (*n* = 16) “sometimes” feel relief, and 10.0% (*n* = 5) “never” feel relief after self-harming. A total of 8% (*n* = 4) of participants reported self-harm after using drugs or alcohol.

A total of 60.0% (*n* = 30) of the participants stated that less than 30 min elapsed between the thought of hurting themselves and carrying out the action, while 40.0% (*n* = 20) stated a more extended period ranging from half an hour to several days or weeks. Regarding the experience of physical pain during self-harm, 46% (*n* = 23) of participants “never” experienced physical pain, 44% (*n* = 22) “sometimes”, and 10.0% (*n* = 5) “always”experienced physical pain during self-harm. Following stressful events, 46% (*n* = 23) of participants “always” self-harm, with failure being the overwhelming situation for 48% (*n* = 24) of the sample, while loss occurred for 20% (*n* = 10), rejection for 18% (*n* = 9), and abandonment for 14% (*n* = 7). In the study sample, 39% (*n* = 19) of adolescents do not try to resist self-harm, while 61% (*n* = 31) use substitute activities (among other things, the use of alcohol and street drugs was also reported) to distract themselves from their urges. More than half of the sample, 56% (*n* = 28), said they had disclosed their behavior to some people, preferably friends (30%, *n* = 15, not listed in Table 1), only 2% (*n* = 4) had disclosed it to most people, while 40% (*n* = 20) said they had not disclosed their behavior to anyone.

The majority of the sample, 78.0% (*n* = 39), stated that the original idea to self-harm was their own, while 22.0% (*n* = 11) stated that they got the idea from the internet/magazines or had seen/heard it from other self-harmers. In the study sample, we found 36% (*n* = 18) of participants were extremely motivated to stop self-harming. A total of 46.0% (*n* = 23) of the participants stated that they did not have any treatment due to self-injury compared to 54.0% (*n* = 27) who had some treatment (individual therapy, medication, family therapy, including self-help). 

The descriptive statistics for the addictive features of NSSI and the mean score ofCraving (C) can be found in Table 2. The mean Craving (C) score was 14.06 ± 7.51. An individual score ≥ 2 on three or more items on a seven-point criterion scale for the addictive features of NSSI was found in 44% of the sample (*n* = 22) (Table 2).

The mean and SD of the OSI functions and the B-SCS score are shown in Table 3. Among the NSSI functions, the Internal ER function score was the highest (18.72 ± 7.08), followed by the External ER function score (8.10 ± 3.11), Social Influence (SI) (5.88 ± 5.37), and Sensation Seeking (3.44 ± 2.98). The mean value of the B-SCS score was 19.54 ± 5.24.

Table 4 shows the mean values of NSSI functions and B-SCS score in relation to suicidal thoughts. Internal ER is statistically significantly different in relation to suicidal thoughts (*p* = 0.041). It was found that there is no statistically significant difference in the values of other NSSI’s functions and B-SCS score in relation to suicidal thoughts (*p* > 0.05) (Table 4).

Regarding the level of motivation to stop self-injuring, we divided the respondents into two groups—extremely motivated and others (“not motivated at all” and “somewhat motivated”). It was found that Internal ER, Sensation Seeking, Craving, and B-SCS score are statistically significantly different regarding the degree of motivation to stop self-injury (*p* = 0.006, *p* = 0.034, *p* = 0.006, respectively) (Table 5). Internal ER, Sensation Seeking, Craving, and B-SCS scores are significantly lower in adolescents who are extremely motivated to stop self-injury (Table 5).

It was found that the B-SCS score is significantly related to Internal ER (r = 0.441, *p* < 0.001) and Craving (r = 0.297, *p* = 0.036) (Table 6).

In a multivariable model that examines the influence of demographic and clinical variables (age, gender, Internal ER, External ER, Sensation Seeking, Craving, cutting, and suicidal thoughts) on B-SCS, it was determined that Internal ER and participants’ age statistically significantly affect the B-SCS score. An increase in Internal ER function and a decrease in age is followed by a higher B-SCS score (*p* = 0.025, i.e., *p* = 0.020) (Table 7).

## 4. Discussion

The present study aimed to investigate the relationship between NSSI and suicidal belief system in adolescent psychiatric inpatients. In the current study, 66% of adolescents reported thoughts of taking their own life in the past year (hereafter referred to as suicidal ideation), and 10% had attempted suicide in the past, which is in line with a large body of evidence of the high rate of suicidal ideation and suicide attempts among NSSI adolescent inpatient [8,9,10,11]. As with other research findings [10,22,23], there was a co-occurrence of NSSI and internalizing and externalizing symptoms in the study sample, with internalizing symptoms being prevalent.

Regarding the descriptive and contextual characteristics of NSSI, the findings of the present study are consistent with findings in related literature in many aspects, including the age of onset of self-injury [2], the most common methods of NSSI [8,23,24,25], the most affected body parts [24], the predominantly distressing urge to self-injure [26], the “impulsive” quality of self-injury in terms of the time that elapses between the thought of self-injury and the performance of the act [24,27], the experience of physical pain during self-injury [24], more of those who self-injure following stressful situations, where failure is considered an overwhelming situation [24], more participants who disclose their self-injurious behavior to others [28], more of participants who engage in substitute acts to avoid self-injury [24]. Although the existing literature on social influence in NSSI talks about contagion or the “spread” of NSSI behavior and reports from one person to another, and that people who have disclosed NSSI (either to adults or peers) are more likely to have friends who self-injure [28], the majority of participants reported that the initial idea to self-injure was their idea. In terms of treatment for NSSI, a similar finding has been reported by other studies in our region, given the higher percentage of adolescents who reported that they had no treatment for NSSI [23,29].

The addictive features of NSSI, characterized by having lost control over the use of NSSI, having built up a notable tolerance to NSSI, and participating in NSSI despite negative consequences [25], were found in 44% of participants in the sample. The mean score for Craving (C) was higher in the present study than in the non-clinical university population [20] and slightly lower than in studies with clinical samples [19], which is consistent with data suggesting that clinical samples may exhibit more addictive features of NSSI than community samples [19].

Consistent with previous studies in clinical samples of adolescents [19,30], our results showed that the most involved functions underlying adolescents’ NSSI behavior are the Internal ER and External ER functions, followed by the reasons for Social Influence and Sensation Seeking functions. In the OSI questionnaire, Internal ER, i.e., the intrapersonal function comprising the function described in the previous review [6], which consists of coping with experiences related to loneliness, sadness, self-punishment, distraction from unpleasant experiences, and interruption of suicidal thoughts and actions [19,20], had the highest score among all other NSSI functions in our study sample.

The B-SCS mean score of 19.54 ± 5.24 obtained in our study was comparable to mean scores in the emergency department and inpatient settings [18], suggesting that maladaptive self-beliefs in the form of unlovable experiences, intolerable emotions, and unsolvable life problems (i.e., suicidal belief system) were significantly prevalent among NSSI inpatient adolescent in our study.

Although this was not the primary focus of this study, it is worth noting that participants with NSSI who reported suicidal ideation had a significantly higher Internal ER function score than those who did not. Victor et al. [11] found that suicidal ideation correlated strongly with the intrapersonal functioning of NSSI to a greater extent than interpersonal functioning. It was hypothesized that individuals struggling with suicidal ideation would use NSSI as a short-term measure to alleviate suicidal ideation, which may lead to greater NSSI engagement [11]. There was a trend toward higher B-SCS score among adolescents who reported suicidal ideation, but no significant differences. The fact that we did not find significant differences in B-SCS scores between the subsamples above in our study may be due to an insufficiently differentiated subsample, which included adolescents with a history of suicide attempts, and should be investigated further with a more differentiated group (suicidal ideation but no suicide attempt and suicidal ideation and suicide attempt).

The results of the study showed that participants who were extremely motivated to stop self-harm had significantly lower scores on Internal ER function and Sensation Seeking, Craving, and B-SCS score than participants who were less or not at all motivated to stop self-harm. To better understand what motivates people to stop NSSI, a previous review [31] suggested that both intra- and inter-personal factors may have an impact on self-harm cessation, including family support, self-esteem, emotional regulation, and professional help.

The current study found a moderate correlation between the B-SCS score, Internal ER function, and Craving. According to Rudd’s notion, suicidogenic beliefs represent a network of interconnected beliefs and thought processes that serve as an individual vulnerability to maladaptive responses to distressing events or experiences [18]. From this perspective, individuals with overly negative beliefs and assumptions about themselves, others, and the world are more likely to experience intense emotions in response to stressful and uncertain life circumstances [32]. Considering the present results, if the thoughts of unsolvability, unlovability, and intolerability are very prevalent and intense in a person’s mind as internalized self-perceptions about one’s worth and ability to endure emotional stress, the person’s overall emotional pain might be more intense when confronted with difficult experiences in life. Difficulty tolerating intense emotions can lead to a tendency to internalize emotional distress, which in turn increases susceptibility to self-harm in these individuals. The strongest relationship between addictive features and Internal ER function among all OSI functions was found in a previous study [26], which discussed that individuals who exhibit Internal ER are more likely to engage in compulsive patterns of NSSI engagement due to their greater psychological vulnerability [26], as individuals who are addicted to NSSI may be notably lacking in available emotional support and the skills to regulate their emotions adaptively. Viewed through a neurodevelopmental perspective, the adolescent period is accompanied by increased emotional intensity and reactivity, so psychophysical changes cause lower tolerance to emotional challenges, less mature emotional regulation, and a greater tendency toward impulsive behavior—due to the dominance of the more mature limbic system, which is responsible for emotional processing, in regard to the less mature prefrontal cortex [33]. Consistent with the previous discussion, the multivariable model showed that Internal ER function and participants’ age related significantly to the B-SCS score. Study findings indicate that decreasing age in our sample of NSSI inpatients predicted higher B-SCS score, showing higher vulnerability embedded in core beliefs about the self as unlovable, one’s emotional experience as unbearable, and life problems as unsolvable (i.e., the suicidal belief system), among younger participants with NSSI. This result deserves attention when it is stated that suicidal ideation is strongly related to age, with the emergence of ideas increasing during early adolescence, with an average age of onset between 10 and 15 years of age [34]. Various cognitions occurring across the fluid suicidal belief system impact the individual’s thoughts, emotions, motivations, and behaviors. That may result in the development and maintenance of NSSI in the presence of challenging and stressful experiences, both intrapersonal and interpersonal. 

## 5. Limitations

The findings of this study should be viewed in the context of its limitations. Due to the small sample size and predominantly female adolescent inpatients, these results may not generalize to other age groups or settings. Given that adolescents can be private about their mental lives, particularly when sharing information with adults, we have to consider the possibility that some of the participants may not have been reliable informants. Using self-report measures, participants may have been subject to various recall and reporting biases. Also, participants were not formally assessed for psychiatric disorders. In addition, due to the cross-sectional data, there was limited ability to make a definitive statement about the causal relationship between the study variables.

## 6. Conclusions

The study results showed that NSSI’s features and function are in line in many aspects with other studies with NSSI adolescent inpatients. Study results showed a high B-SCS score among NSSI adolescent inpatients, indicating a high prominence of suicidogenic cognition comprising the suicidal belief system. Our model showed that Internal ER function and participants’ age related significantly to the B-SCS score. Although cognitions occurring across the fluid suicidal belief system alone do not fully capture the complexity of suicide, assessing the suicidal belief system in NSSI inpatient adolescents could nevertheless provide helpful information for identifying individuals who may have an elevated vulnerability to experiencing suicidal ideas and behaviors over time. In addition to increasing the knowledge on these topics, gaining a deeper understanding of self-injuring behaviors in adolescents is essential to developing more sophisticated and comprehensive treatment methods.

## Figures and Tables

**Table 1:** NSSI’s descriptive and contextual features.

OSI Items	Count (*n*)	%
How often in the past 6 months have you actually injured yourself. without the intention to kill yourself?		
Not at all	0	0.0
1–5 times	4	8.0
Monthly	14	28.0
Per week	28	56.0
Daily	4	8.0
Have thought about taking your life in the past year	33	66.0
Ever made an actual attempt to take your life	5	10.0
Been treated by a doctor after injuring yourself on purpose	10	20.0
Stay overnight in emergencu	2	4.0
How old were you when you started to self-injure?	12.96 ± 1.63	9–17 years
The first time you hurt yourself. where did you get idea		
Read about it on an internet website	5	10.0
Read about it in a book or magazine	1	2.0
I saw other people do it in a hon-hospital setting	3	6.0
I heard about it from other people in a hospital setting	2	4.0
It was my own idea	39	78.0
When you get urge to hurt yourself		
The urge is distressing/upsetting	2.14 ± 1.41	0–4
The urge is comforting	1.64 ± 1.35	0–4
The urge is intrusive/invasive	2.16 ± 1.38	0–4
Only harm yourself after taking drugs and alcohol	4	8.0
Da you let other people know you harm yourself		
no one	20	40.0
some people (friend. psychologist/psychiatris)	28	56.0
most people	2	4.0
What areas of your body did/do you injure? Currently		
face	9	18.0
lower arm/wrist	30	60.0
thigh/knee	26	52.0
abdomen	10	20.0
hand/fingers	14	28.0
How did you injure yourself (without meaning to kill yourself) Currently		
cutting	24	48.0
scratching	12	24.0
piercing skin with sharp objects	9	18.0
taking too much medication	2	4.0
biting	7	14.0
Do you feel relief after harming yourself?		
never	5	10.0
sometimes	16	32.0
always	29	58.0
Once you think about harming yourself. do you always do?		
Yes	11	22.0
No	39	78.0
How much time goes by between thinking about it and doing it?	Frequency	Percent
less than 1 min	2	4.0
1 minute to 5 min	11	22.0
6–30 minutes	17	34.0
over 30 min but less than 1 h	11	22.0
hours	4	8.0
days	5	10.0
Do you feel physical pain when you harm yourself?		
never	23	46.0
sometimes	22	44.0
always	5	10.0
Do you hurt or think about hurting yourself after stressful thinks happen?		
never	8	16.0
sometimes	19	38.0
always	23	46.0
Stressful situation typically lead to self injury?		
failure	24	48.0
rejection	9	18.0
loss	10	20.0
abandonment	7	14.0
If you are trying to resist hurting yourself. what do you do instead?		
never try to resist	19	38.0
talk with someone	12	24.0
exercise/sports	2	4.0
reading. writing. music. dance....	5	10.0
watch television. play video or computer games	4	8.0
do things to relax...	5	10.0
use alcohol or street drogs	1	2.0
do anything to keep hands busy	2	4.0
How motivated are you at this time to stop self injury?		
not at all	9	18.0
somewhat	23	46.0
extremely	18	36.0
What treatmen have you recived with the goal of reducing and/or eliminating your selfharm		
not had a treatment	23	46.0
individual therapy	10	20.0
family therapy	4	8.0
medication	8	16.0
self help	5	10.0

**Table 2:** NSSI’s addictive features.

	OSI Items		Never		Sometimes	Always	
			0	1	2	3	4
1	The self injurious behavior occurs often than intended	*n*	16	8	18	7	1
		%	32.0	16.0	36.0	14.0	2.0
2	Self injurious behavior occures has increased	*n*	10	6	11	16	7
		%	20.0	12.0	22.0	32.0	14.0
3	The need to self injurious themselves more frequently or greater intensity to produce the same effect	*n*	16	6	8	8	12
		%	32.0	12.0	16.0	16.0	24.0
4	The behavior thinking about it consumes a significant amount of time	*n*	16	4	10	11	9
		%	32.0	8.0	20.0	22.0	18.0
5	Despite a desire to cut down or control this behavior you are unable to do so	*n*	13	7	9	7	14
		%	26.0	14.0	18.0	14.0	28.0
6	Continue this behavior despite recognizing that is harmful to you/psysically end/or emotionally	*n*	6	5	8	9	22
		%	12.0	10.0	16.0	18.0	44.0
7	Important social. family. academic or recreational are given up or reduced because of this behavior	*n*	12	5	10	12	11
		%	24.0	10.0	20.0	24.0	22.0
	Craving (C)	14.06 ± 7.51			0–24		
	Three/more YES responses	22.0			44.0		

**Table 3:** NSSI’s function scores and B-SCS score.

OSI Functions	Min Score	Max Score	Mean ± SD
Internal ER	0	28	18.72 ± 7.08
Social Influence	0	20	5.88 ± 5.37
External ER	0	12	8.10 ± 3.11
Sensation Seeking	0	14	3.44 ± 2.98
Craving	0	24	14.06 ± 7.51
B-SCS	6	30	19.54 ± 5.24

**Table 4:** NSSI’s function scores and B-SCS score in relation to suicidal thoughts.

OSI Functions	Suicidal Thoughts		*p* ^1^
	Yes	No	
Internal ER	20.18 ± 6.18	15.88 ± 7.99	0.041
Social Influence	6.30 ± 5.66	5.09 ± 4.79	0.452
External ER	8.54 ± 2.89	7.23 ± 3.40	0.195
Sensation Seeking	3.55 ± 2.98	3.44 ± 2.98	0.685
Craving	14.64 ± 7.02	12.94 ± 8.48	0.525
B-SCS	20.36 ± 5.24	17.94 ± 5.02	0.162

^1^ Mann-Whitney test.

**Table 5:** NSSI’s functions scores and B-SCS score in relation to motivation to stop self-injuring.

OSI Functions	Motivation to Stop Self-Injuring		*p* ^1^
	Others	Extremely Motivated	
Internal ER	20.87 ± 6.41	14.89 ± 6.72	0.006
SocialInfluence	6.06 ± 6.04	5.55 ± 4.07	0.776
External ER	8.69 ± 2.86	7.06 ± 3.33	0.075
SensationSeeking	4.09 ± 3.09	2.27 ± 2.44	0.034
Craving	16.31 ± 6.46	10.06 ± 7.72	0.006
B-SCS	20.91 ± 4.29	17.11 ± 5.89	0.017

^1^ Mann-Whitney test.

**Table 6:** Correlations among NSSI’s function scores and B-SCS score.

		Internal ER	Social Influens	External ER	Sensation Seeking	Craving
B-SCS total	r	441 **	0.118	0.191	0.171	0.297 *
	*p*	0.001	0.413	0.183	0.236	0.036
Internal ER	r	1	0.406 **	0.551 **	0.531 **	0.648 **
	*p*		0.003	0.000	0.000	0.000
Social Influence	r		1	0.343 *	0.455 **	0.429 **
	*p*			0.015	0.001	0.002
External ER	r			1	0.535 **	0.668 **
	*p*				0.000	0.000
Sensation Seeking	r				1	0.725 **
	*p*					0.000

r—correlation coefficient, * *p* < 0.05.** *p* < 0.01.

**Table 7:** Assessment of the impact of factors of interest on the BSC-S score, the results of the multivariate linear regression analysis.

	B	95.0% Confidence Interval for B		Beta	*p*	Tolerance	VIF
Age	−1.273	−2.336	−0.211	−0.337	0.020	0.806	1.241
Gender	−0.105	−3.780	3.570	−0.008	0.954	0.861	1.161
Internal ER	0.346	0.046	0.647	0.468	0.025	0.386	2.591
Social Influence	−0.040	−0.330	0.250	−0.041	0.782	0.724	1.381
External ER	−0.402	−1.018	0.214	−0.238	0.194	0.479	2.089
Sensation Seeking	−0.513	−1.185	0.159	−0.292	0.131	0.436	2.293
Craving	0.282	−0.034	0.599	0.404	0.079	0.310	3.224
cutting	0.481	−2.621	3.583	0.046	0.756	0.715	1.399
suicidal thoughts	1.823	−1.234	4.881	0.166	0.235	0.818	1.222
(Constant)	32.761	16.100	49.422		0.000		

Model adjusted R^2^ = 0.236, *p* = 0.015, B—unstandardized regression coefficient, Beta—standardized regression coefficient, 95% CI—95% confidence interval.

## Data Availability

Data generated and analyzed during the study are available upon request to the author.
